# Parental predictors of an Internet-based parenting intervention for child disruptive behavior: an implementation study

**DOI:** 10.1007/s00787-025-02928-x

**Published:** 2025-11-28

**Authors:** Yujing Li, Amit Baumel, Susanna Hinkka-Yli-Salomäki, Malin Kinnunen, Terja Ristkari, Minja Westerlund, Andre Sourander

**Affiliations:** 1 Department of Child Psychiatry, Research Centre for Child Psychiatry, University of Turku, Lemminkäisenkatu 3a, Turku, 20014 Finland; 2 Finland INVEST Research Flagship, University of Turku, Turku, Finland; 3 Department of Community Mental Health, University of Haifa, Haifa, Israel; 4 Department of Child Psychiatry, Turku University Hospital, Turku, Finland

**Keywords:** Child disruptive behavior, Implementation, Internet-based intervention, Predictors, Parenting

## Abstract

This study investigates parental factors as predictors of the outcome of an internet-based and telephone-assisted parent training intervention targeting child disruptive behavior, when it was implemented nationwide in Finland. 2,900 families with children who met screening criteria at their 4-year-old health checkup received the 11-week intervention. Potential predictors included parents’ age and educational attainment, family structure, enrollment year, parenting skills, and parental mental health. The outcome was the Child Behavior Checklist 1.5-5 externalizing score, collected at baseline, 6-, 12-, and 24-month follow-ups. Hierarchical linear models were used to examine changes in the outcome over time. At 6 months, parents of children whose mothers had a college or university degree reported smaller reductions in child externalizing problems compared to those with lower maternal education (*β*= 0.87, 95% CI [0.28, 1.45], *p*= .004). At 24 months, greater reductions in externalizing problems were observed in children whose parents had a high parental over-reactivity score at baseline (*β*= -1.23, 95% CI [-1.97, -0.49], *p*= .001), as well as a medium or high baseline parental Depression, Anxiety and Stress Scale total score (Medium: *β*= -1.01, 95% CI [-1.65, -0.36], *p*= .002; High: *β*= -1.05, 95% CI [-1.82, -0.28], *p*= .007). There were greater changes in the outcome over time among disadvantaged populations, particularly families with lower education, poorer parenting skills, or more severe parental mental health problems. Future research is needed to examine the consistency of these effects across diverse sociodemographic groups and settings.

**Supplementary Information:**

The online version contains supplementary material available at 10.1007/s00787-025-02928-x.

## Introduction

Child disruptive behavior refers to a range of externalizing behaviors, including aggression, defiance, hyperactivity, and rule-breaking, that are associated with poor academic outcomes [1], difficulty in forming social relationships [2, 3], as well as an increased risk of later mental health problems [3, 4], substance use disorder [3] and criminal activity ​ [3–5]. Parent training interventions are a well-established and effective approach for treating child disruptive behavior [6, 7]. These interventions focus on enhancing parenting skills to promote positive parent–child interactions, improve child behavior, and alleviate family stress [8]. Evidence-based programs, such as *The Incredible Years* and *Triple P – Positive Parenting Program*, have consistently proven effective in reducing externalizing problems and enhancing parenting practices in randomized controlled trials (RCTs) [9, 10]. More recently, digital parent training has emerged as a promising alternative, which offers a more affordable and accessible approach for broader populations. Meta-analyses showed these digital programs reduce child externalizing problems, with outcomes comparable to face-to-face delivery [11, 12].

It has been suggested that digital parent training interventions can provide substantial public health benefits if implemented at scale [13]; however, large-scale implementation remains limited and challenging. Understanding how intervention outcomes vary across diverse family contexts and for whom an intervention works (that is, identifying predictors of intervention outcomes) can help better target families that are most likely to benefit and guide the development of tailored programs for a broader range of families [14]. Parental factors, including age, education, family structure, parenting practices, and mental health, have been examined as potential predictors of parenting programs, with mixed findings reported. Some studies showed that younger maternal age and higher educational attainment predict larger improvements [14, 15], while others found no associations [16, 17] or even the opposite [18]. Similarly, high levels of harsh parenting practices and poor parental mental health have been associated with poorer treatment outcomes in some studies [15, 17] but not consistently across samples [16, 19, 20]. These inconsistencies highlight the complexity of predicting intervention outcomes and underscore the need for further investigation. Moreover, prior studies were typically conducted in RCT or clinical settings. Complementing RCT analyses with larger samples from routine settings is important for extending findings to real-world contexts and populations. Additionally, research has primarily focused on traditional face-to-face or group-based interventions, with fewer studies on digital formats [16, 18].

The Finnish Strongest Families Parenting (FSFP) intervention is an internet-based and telephone-assisted parent training program, targeting child disruptive behavior, that has been successfully implemented in Finland [21]. The program is grounded in social learning theory, consisting of 11 sessions that focus on developing parenting skills to improve parent–child relationships, encourage positive behavior, manage routine adjustments, prepare for challenging situations, and promote pro-social behavior [22]. A previous RCT of the same intervention showed greater improvements in the intervention group at 24-month follow-up [23]. In a subsequent implementation study using the same FSFP intervention, no significant differences were observed between the implementation and RCT intervention groups regarding Child Behavior Checklist (CBCL) externalizing scores at 6-month follow-up [24]. In the present study, we aim to identify parental factors that predict the intervention outcome, thereby identifying individuals who benefit most, using an implementation study design with a large sample size and a two-year follow-up. Specifically, we investigated the potential predictive roles of parental factors including maternal and paternal age, education, family structure, parenting skills, and mental health, as well as the enrollment year.

## Methods

### Study design

**Fig. 1:**
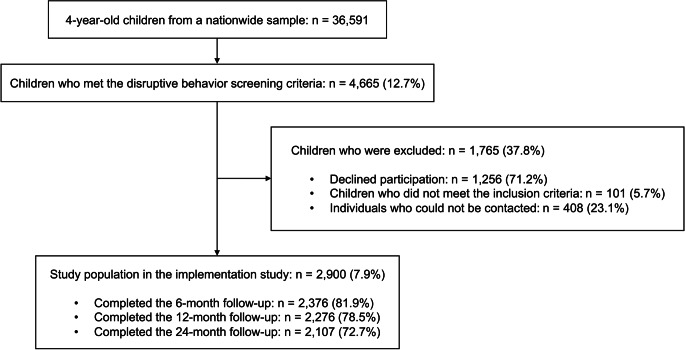
Flow chart of study population selection

The implementation study consisted of 2,900 families who received the FSFP intervention between January 2015 and December 2021 when it was implemented in real-world practice in child health clinics in 16 administrative regions across Finland. Within one month of their child reaching the age of 4, each family received a study information package and was instructed to complete a health questionnaire, which they were requested to bring to their child’s annual health check-up. The FSFP intervention was introduced to families during the health check-up, with those whose children screened positive for disruptive behavior being contacted remotely thereafter. The conduct problems subscale of the Strengths and Difficulties Questionnaire (SDQ) was used for screening, with a score of ≥ 5, representing the 80th percentile cutoff based on a Finnish study of 4-year-olds (*n* = 931) [25], to identify eligible participants. Additional criteria for inclusion and exclusion were described in the Supplementary Material. Children were eligible for the study if they met the criteria and their parents provided informed consent. During the initial screening, 36,591 children were assessed for symptoms of highly disruptive behavior, with 4,665 (12.7%) meeting the eligibility criteria. Figure 1 presents the participant flow throughout the study.

Ethical approval for the implementation was granted by the University of Turku (approval number: 18/2018).

## Procedures

The FSFP program integrates an interactive website with weekly telephone coaching [21]. Trained coaches conducted weekly telephone consultations with parents and continuously monitored their progress via the program website. The program consisted of 11 weekly themes. The first 7 sessions focused on teaching positive and practical parenting strategies, including positive problem-solving skills and understanding the child’s emotional development. Later sessions focused on applying these skills in daily life and maintaining them after the program. The content and the framework of the weekly themes are depicted in Table S1. Children were not involved in the web interactions or coaching calls.

Details regarding the intervention procedures, quality assurance, and implementation strategies are presented in the Supplementary Material.

## Measures

Demographic characteristics (i.e., mother’s age at birth, father’s age at birth, maternal education, paternal education, and family structure) were reported by the responsible parent at baseline. The date of enrollment was recorded accordingly. Baseline parenting skills were evaluated using the 30-item Parenting Scale [26, 27]. The Cronbach’s alpha coefficients for the 5-item Laxness subscale, the 5-item Over-reactivity subscale, and the 3-item Hostility subscale in our study were 0.65, 0.66, and 0.48, respectively. The relatively low *α* of the Hostility subscale may be attributed to the small number of items. Baseline parental mental health was assessed using the 21-item short form of the Depression, Anxiety and Stress Scale (DASS-21) [28, 29]. In our sample, the DASS total score demonstrated excellent internal consistency (Cronbach’s *α* = 0.91). The outcome of this study was the externalizing scale of the 24-item CBCL 1.5–5.5 (CBCL/1.5–5.5) [30], which was assessed at baseline and at 6-month, 12-month, and 24-month follow-ups. The Cronbach’s alpha coefficient for the CBCL externalizing score in this sample was 0.87.

## Statistical analyses

Hierarchical linear mixed-effects models were used to investigate whether the parental factors predicted changes in child externalizing problems over time in the FSFP intervention, across repeated measurements at baseline and 6-, 12-, and 24-month follow-ups. Spearman’s rank correlation and Cramér’s V were used to calculate the correlations between ordinal-ordinal and nominal-ordinal variables, respectively (Fig. S1). Most variables were not correlated; only maternal and paternal age (*r* =.55) and maternal and paternal education (*r* =.43) showed moderate correlations. Missing data were addressed using maximum likelihood estimation. In each model, time was specified as the within-subject factor, while the potential predictor and its interaction with time served as the between-subject factors. The models were adjusted for all other parental factors except the predictor of interest. To mitigate multicollinearity, continuous variables (i.e., the Parenting Scale subscale scores and the DASS total score) were standardized through *z*-transformation. The *z*-transformed scores were further categorized into three groups: low (*z* < −0.5), medium (−0.5 ≤ *z* ≤ 0.5), and high (*z* > 0.5). All analyses were conducted using R 4.4.1. A two-tailed *p*-value of less than 0.05 was considered statistically significant.

## Results

### Baseline characteristics

Table 1 presents the baseline characteristics of the 2,900 participants, while Fig. S2 and Fig. S3 illustrate trends in some characteristics over the enrollment years. Both the baseline CBCL externalizing score and the baseline parental DASS total score showed an increase across the enrollment years. Table S2 presents the baseline characteristics of participants retained at 24-month follow-up compared with those lost to follow-up.

**Table 1:** Baseline characteristics of the participants

	FSFP implementation (*n* = 2900)
Child characteristics	
Sex^a^, *n* (%)	
Male	1828 (63.3)
Female	1062 (36.7)
Baseline CBCL externalizing score, mean (SD)	21.7 (0.1)
Parent and family characteristics	
Maternal age at birth^b^, mean (SD)	30.4 (5.0)
< 25 years, n (%)	345 (12.0)
25–35 years, n (%)	2090 (72.6)
> 35 years, n (%)	442 (15.4)
Paternal age at birth^c^, mean (SD)	32.6 (5.7)
< 25 years, n (%)	189 (6.8)
25–35 years, n (%)	1805 (65.1)
> 35 years, n (%)	777 (28.1)
Maternal education^d^, n (%)	
Secondary education or less	1036 (35.9)
College or university degree	1851 (64.1)
Paternal education^e^, n (%)	
Secondary education or less	1427 (52.3)
College or university degree	1302 (47.7)
Family structure^f^, n (%)	
Two biological parents	2389 (82.7)
One biological parent and other structures	500 (17.3)
Enrollment year, n (%)	
2015–2017	707 (24.4)
2018–2019	1081 (37.3)
2020–2021	1112 (38.3)
Parenting Scale Laxness score^g^, n (%)	
Low	1020 (35.2)
Medium	1094 (37.7)
High	785 (27.1)
Parenting Scale Over-reactivity score^h^, n (%)	
Low	856 (29.5)
Medium	1147 (39.6)
High	896 (30.9)
Parenting Scale Hostility score^i^, n (%)	
Low	1377 (47.5)
Medium	676 (23.3)
High	846 (29.2)
DASS Total score^j^, n (%)	
Low	1096 (37.8)
Medium	1153 (39.8)
High	650 (22.4)

*FSFP* Finnish Strongest Families Parenting, *SD* standard deviation, *DASS* the 21-item short form of the Depression, Anxiety and Stress Scale

^a^Missing observations: 10; ^b^Missing observations: 23; ^c^Missing observations: 129; ^d^Missing observations: 13;

^e^Missing observations: 171; ^f^Missing observations: 11; ^g^Missing observations: 1; ^h^Missing observations: 1; ^i^Missing observations: 1; ^j^Missing observations: 1.

### Predictors of changes in the outcome

**Fig. 2:**
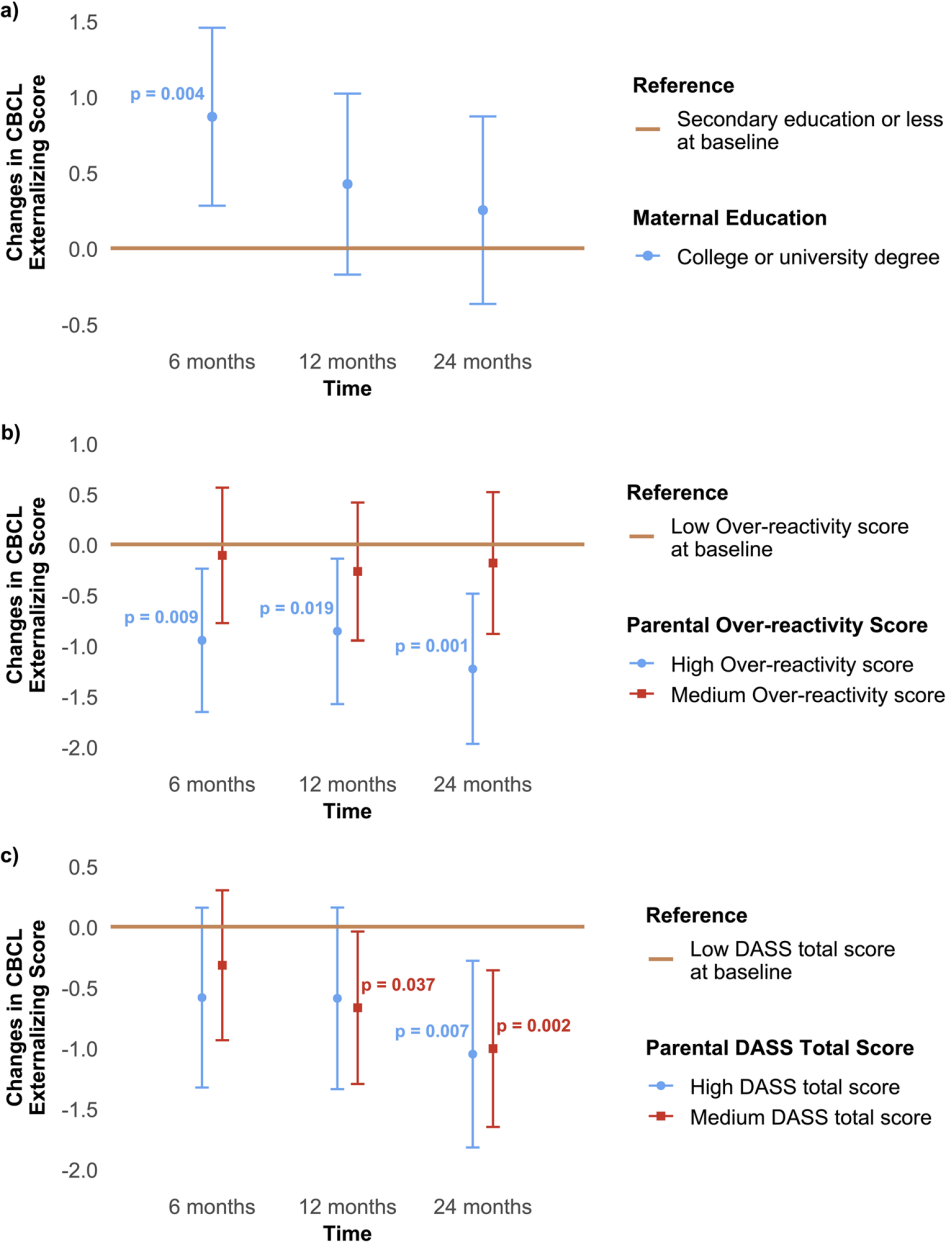
Changes in Child Behavior Checklist 1.5–5.5 (CBCL) externalizing scores over time by (**a**) maternal education, (**b**) baseline parental Over-reactivity scores, and (**c**) baseline parental Depression, Anxiety and Stress Scale (DASS) total score. Note: The horizontal line represents the corresponding reference group at baseline. A positive change indicates a smaller reduction in the CBCL externalizing score (i.e., less improvement) compared to the baseline reference group, while a negative change indicates a greater decrease in the score (i.e., better improvement) compared to the reference at baseline. Only statistically significant *p*-values (< 0.05) are displayed in the figure

The mean CBCL externalizing score decreased over time, from 21.7 at baseline to 16.1 at 6 months, 15.6 at 12 months, and 14.4 at 24 months. Tables 2 and 3; Fig. 2 provide the results of the hierarchical linear models. A positive coefficient indicates that the decline in scores was smaller in that subgroup compared with the reference group, and vice versa. Higher maternal education was associated with a smaller decrease in CBCL externalizing scores over time (*p* =.033), while higher over-reactivity score (*p* =.016) and DASS total score at baseline (*p* =.040) were linked to a greater decrease in child externalizing problems, compared to their respective reference groups. Notably, while the predictive effect of maternal education diminished over time (*β* = 0.87, 95% CI [0.28, 1.45], *p* =.004 at 6 months; *β* = 0.25, 95% CI [−0.37, 0.87], *p* =.426 at 24 months), the predictive effects of over-reactivity score and DASS total score became more pronounced, with progressively greater reductions in CBCL externalizing scores observed at 6, 12, and 24 months relative to baseline (High over-reactivity score: *β* = −0.95, 95% CI [−1.66, −0.24], *p* =.009 at 6 months; *β* = −1.23, 95% CI [−1.97, −0.49], *p* =.001 at 24 months. Medium DASS total score: *β* = −0.32, 95% CI [−0.94, 0.30], *p* =.313 at 6 months; *β* = −1.01, 95% CI [−1.65, −0.36], *p* =.002 at 24 months. High DASS total score: *β* = −0.58, 95% CI [−1.33, 0.16], *p* =.122 at 6 months; *β* = −1.05, 95% CI [−1.82, −0.28], *p* =.007 at 24 months).

The severity of child disruptive behaviors was assessed using the baseline SDQ conduct score to explore its potential role in the predictive effects of maternal education on CBCL externalizing scores. At baseline, children of mothers with a college or university degree had significantly lower SDQ conduct scores (mean ± SD: 6.17 ± 1.26) compared to those whose mothers had a secondary or lower degree (6.32 ± 1.34; Wilcoxon test, *p* =.005).

Parental over-reactivity scores declined over time, decreasing from 3.83 ± 0.96 at baseline to 3.13 ± 0.95 at 6-month follow-up. However, the effect size of this reduction was greater among children whose parents had high baseline over-reactivity scores (Cohen’s *d* = 1.28) compared to those with medium (*d* = 0.93) or low baseline scores (*d* = 0.31). Similarly, the mean parental DASS total score declined from 20.2 ± 15.9 at baseline to 13.6 ± 13.3 at 6 months. The reduction was greater among those with high levels of parental mental health problems at baseline (*d* = 0.92) than those with medium (*d* = 0.59) or low (*d* = 0.004) levels.

The interactions between time and maternal age, paternal age, paternal education, family structure, enrollment year, laxness score, and hostility score were not significant, showing that these variables did not exhibit time-dependent predictive effects on the intervention outcome.

**Table 2:** Hierarchical linear models results for parental demographic factors

CBCL externalizing	B	SE	95% CI	*P* value	Overall *P* value
Maternal age at birth					0.122
Intercept	18.44	0.47	(17.51, 19.37)	< 0.001	
< 25 years × 6 months	−1.29	0.47	(−2.20, −0.40)	0.005	
< 25 years × 12 months	−0.72	0.48	(−1.65, 0.22)	0.134	
< 25 years × 24 months	−1.20	0.50	(−2.17, −0.23)	0.016	
>35 years × 6 months	−0.32	0.40	(−1.10, 0.46)	0.425	
>35 years × 12 months	0.01	0.40	(−0.78, 0.79)	0.987	
>35 years × 24 months	−0.27	0.41	(−1.08, 0.54)	0.514	
Paternal age at birth					0.304
Intercept	18.60	0.48	(17.67, 19.54)	< 0.001	
< 25 years × 6 months	−0.92	0.61	(−2.11, 0.28)	0.133	
< 25 years × 12 months	−0.31	0.63	(−1.55, 0.92)	0.619	
< 25 years × 24 months	−0.22	0.65	(−1.49, 1.05)	0.734	
>35 years × 6 months	0.42	0.32	(−0.20, 1.04)	0.182	
>35 years × 12 months	0.58	0.32	(−0.05, 1.21)	0.073	
>35 years × 24 months	0.54	0.33	(−0.11, 1.19)	0.102	
Maternal education					0.033
Intercept	18.76	0.48	(17.81, 19.71)	< 0.001	
College or university degree × 6 months	0.87	0.30	(0.28, 1.45)	0.004	
College or university degree × 12 months	0.42	0.30	(−0.17, 1.02)	0.165	
College or university degree × 24 months	0.25	0.32	(−0.37, 0.87)	0.426	
Paternal education					0.263
Intercept	18.66	0.48	(17.72, 19.59)	< 0.001	
College or university degree × 6 months	0.51	0.28	(−0.04, 1.06)	0.047	
College or university degree × 12 months	0.43	0.28	(−0.13, 0.99)	0.100	
College or university degree × 24 months	0.24	0.29	(−0.33, 0.81)	0.325	
Family structure					0.652
Intercept	18.52	0.47	(17.60, 19.45)	< 0.001	
One biological parent and other structures × 6 months	0.04	0.42	(−0.79, 0.86)	0.929	
One biological parent and other structures × 12 months	0.15	0.43	(−0.70, 0.99)	0.734	
One biological parent and other structures × 24 months	−0.41	0.44	(−1.28, 0.46)	0.352	
Enrollment year					0.075
Intercept	18.78	0.49	(17.82, 19.74)	< 0.001	
2018–2019 × 6 months	0.79	0.38	(0.05, 1.52)	0.036	
2018–2019 × 12 months	−0.08	0.38	(−0.83, 0.67)	0.840	
2018–2019 × 24 months	0.63	0.38	(−0.11, 1.37)	0.095	
2020–2021 × 6 months	1.00	0.38	(0.26, 1.74)	0.008	
2020–2021 × 12 months	−0.03	0.38	(−0.78, 0.72)	0.944	
2020–2021 × 24 months	0.73	0.38	(−0.02, 1.49)	0.056	

Models were adjusted for baseline covariates, including maternal age at birth, paternal age at birth, maternal education, paternal education, family structure, enrollment year, Parenting Scale Laxness, Over-reactivity, and Hostility scores, and Depression, Anxiety and Stress Scale total score, except the predictor of interest. *CBCL externalizing* Child Behavior Checklist 1.5–5.5 externalizing scale; *SE* standard error, *95% CI* 95% confidence interval

**Table 3:** Hierarchical linear models results for parental psychological factors

CBCL externalizing	B	SE	95% CI	*P* value	Overall *P* value
Parenting Scale Laxness score					0.423
Intercept	18.66	0.48	(17.72, 19.61)	< 0.001	
Medium × 6 months	0.66	0.33	(0.02, 1.30)	0.044	
Medium × 12 months	0.51	0.33	(−0.14, 1.16)	0.126	
Medium × 24 months	0.46	0.34	(−0.20, 1.13)	0.173	
High × 6 months	0.01	0.36	(−0.69, 0.72)	0.972	
High × 12 months	−0.05	0.36	(−0.76, 0.66)	0.886	
High × 24 months	0.00	0.38	(−0.73, 0.74)	0.994	
Parenting Scale Over-reactivity score					0.016
Intercept	18.28	0.49	(17.33, 19.23)	< 0.001	
Medium × 6 months	−0.11	0.34	(−0.78, 0.56)	0.750	
Medium × 12 months	−0.27	0.35	(−0.95, 0.41)	0.441	
Medium × 24 months	−0.18	0.36	(−0.89, 0.52)	0.606	
High × 6 months	−0.95	0.36	(−1.66, −0.24)	0.009	
High × 12 months	−0.86	0.37	(−1.58, −0.14)	0.019	
High × 24 months	−1.23	0.38	(−1.97, −0.49)	0.001	
Parenting Scale Hostility score					0.984
Intercept	18.53	0.48	(17.59, 19.47)	< 0.001	
Medium × 6 months	0.08	0.35	(−0.61, 0.77)	0.830	
Medium × 12 months	−0.07	0.36	(−0.77, 0.63)	0.842	
Medium × 24 months	0.08	0.37	(−0.64, 0.80)	0.828	
High × 6 months	−0.17	0.34	(−0.82, 0.49)	0.621	
High × 12 months	0.08	0.34	(−0.59, 0.75)	0.817	
High × 24 months	−0.00	0.35	(−0.69, 0.68)	0.993	
DASS Total score					0.040
Intercept	18.25	0.48	(17.31, 19.20)	< 0.001	
Medium × 6 months	−0.32	0.32	(−0.94, 0.30)	0.313	
Medium × 12 months	−0.67	0.32	(−1.30, −0.04)	0.037	
Medium × 24 months	−1.01	0.33	(−1.65, −0.36)	0.002	
High × 6 months	−0.58	0.38	(−1.33, 0.16)	0.122	
High × 12 months	−0.59	0.38	(−1.34, 0.16)	0.122	
High × 24 months	−1.05	0.39	(−1.82, −0.28)	0.007	

Models were adjusted for baseline covariates, including maternal age at birth, paternal age at birth, maternal education, paternal education, family structure, enrollment year, Parenting Scale Laxness, Over-reactivity, and Hostility scores, and DASS total score, except the predictor of interest. *CBCL externalizing* Child Behavior Checklist 1.5–5.5 externalizing scale; *SE* standard error; *95% CI* 95% confidence interval; *DASS* the 21-item short form of the Depression, Anxiety and Stress Scale

## Discussion

This study examined whether various parental factors and enrollment year predict changes in the outcome of an internet-based and telephone-assisted parent training program implemented in Finland. Maternal education, parenting skills, and parental mental health were found to predict changes in the intervention outcomes over time. This study investigated predictors of a digital intervention for child disruptive behavior within a real-world implementation setting, rather than in RCT or clinical contexts. The focus on digital interventions, as opposed to traditional face-to-face or group-based interventions, addresses the scarcity of research in this field. Additionally, our study extends previous research by incorporating a longer follow-up period of 2 years, providing a more comprehensive view of the predictive effects of the intervention. Moreover, the large sample size of 2,900 families offers a broader and more diverse population for analysis, as well as greater statistical power.

Mothers with a college or university degree were more likely to report less improvement in CBCL externalizing problems following the intervention, suggesting that mothers with lower education were more responsive to the intervention compared to their more highly educated counterparts. Notably, the predictive effect of maternal education diminished over time (i.e., a significant difference of 0.87 in CBCL externalizing scores at 6 months and a non-significant difference of 0.25 at 24 months). Given findings from previous RCTs showing that the FSFP intervention effects persisted for up to 24 months [23, 31], we suggest that the intervention may provide comparable benefits to parents across different levels of education in the long term. Conversely, previous research has reported that children whose mothers had a university degree showed greater reductions in disruptive behavior to a parent training program implemented nationwide in Sweden [14]. One possible explanation for our finding is that children of highly educated mothers exhibited less severe behavioral difficulties at baseline, limiting the potential for improvement. This is supported by our findings that mothers with a college or university degree had children with significantly lower baseline SDQ conduct scores (mean ± SD: 6.17 ± 1.26) compared to those with a secondary or lower degree (6.32 ± 1.34). Another possibility is that highly educated mothers may already use more effective parenting strategies, leaving less room for improvement.

Parental over-reactivity score at baseline was a predictor of changes in the intervention outcome, whereas parental laxness and hostility scores did not exhibit any predictive effects. Specifically, parents with higher baseline over-reactivity scores showed a greater decrease in CBCL externalizing scores compared to those with lower scores. This finding indicates that parents exhibiting a harsher, more impulsive, or exaggerated parenting style at baseline experienced more substantial improvements in reducing child externalizing problems. One core technique of the FSFP program focuses on reducing impulsive or exaggerated parental responses and replacing them with more positive approaches, addressing the underlying dynamics driving over-reactive parenting styles. In the implementation sample, over-reactivity scores decreased over time, whilst the effect size of this reduction was larger for those with high over-reactivity scores at baseline compared to those with medium or low over-reactivity scores at baseline. These analyses validated our hypothesis that parents with higher over-reactivity scores (i.e., poorer parenting skills) had greater potential for improvement by practicing the strategies provided by the intervention in response to challenging situations.

Similarly, families with higher levels of harsh and inconsistent parenting were found to have greater reductions in child externalizing problems following *The Incredible Years* program [20]. However, an RCT identified no significant predictive effects of parenting behaviors and parental self-efficacy on the effectiveness of an internet-based intervention for child disruptive behavior [16]. The heterogeneity in results and the notable lack of studies examining parenting skills/styles as potential predictors, particularly in the context of child disruptive behavior and digital interventions, underscore the critical need for further research.

Parental mental health also emerged as a predictor of intervention outcomes. Higher parental DASS total scores at baseline were associated with greater reductions in CBCL externalizing scores at 6-, 12-, and 24-month follow-ups compared to low DASS total scores. These findings are somewhat unexpected, as prior studies have reported contradictory findings: some showed that higher levels of maternal stress or distress predicted poorer treatment outcomes [17, 32], while other studies found no predictive effect of maternal stress or depression [16, 19]. Inconsistencies in findings may arise from heterogeneity in study populations, measurement approaches, or the intervention evaluated. One plausible explanation of our findings is that parents with poorer mental health may initially exhibit more negative parent–child interactions and less effective parenting practices [33], offering more scope for improvements following the intervention. Alternatively, the intervention may equip these parents with strategies to better manage stress and regulate responses, which, in turn, foster more positive parent–child dynamics and improved child outcomes. These positive interactions may also be rewarding for parents, encouraging sustained engagement, particularly for those with depressive symptoms, who might otherwise experience less positive feedback. Additionally, parents with poorer mental health may be less attuned to, or less likely to report, their child’s disruptive behaviors, contributing to the observed declines in externalizing scores.

Notably, children of parents with poorer baseline mental health showed a progressively greater reduction in CBCL externalizing scores over time (from − 0.58 at 6 months to −1.05 at 24-month follow-up). Meanwhile, parents with high levels of mental health problems at baseline experienced greater reductions in DASS total score over time than those with medium or low levels. The observed patterns suggest a potential benefit of the intervention on parental mental health, contributing to sustained improvement in child behaviors. Parents exhibiting high baseline mental health difficulties may receive additional psychosocial support through the intervention, which could facilitate more positive interactions with their child. These rewarding experiences may, in turn, enhance parental mental health and their motivation to engage in positive parenting practices. The virtuous circle may partly explain the predictive effect over time.

In this study, no predictive effects were observed for maternal age, paternal age, paternal education, family structure, or enrollment year, indicating that the intervention was broadly applicable and equally delivered across the population. Similarly, maternal age was not found to predict treatment outcomes in a Norwegian study [17]. Our findings regarding family structure align with previous research, which reported no significant effect of family structure on the intervention’s impact on child externalizing problems [16, 17]. However, there is limited direct evidence examining fathers’ characteristics as predictors; most have focused on maternal or parental demographics, which complicates direct comparisons with our findings.

Although the overall predictive effect of enrollment year was not significant, children enrolled in 2020–2021 showed a significant increase in CBCL externalizing scores at 6-month follow-up compared to those enrolled in 2015–2017. This suggests that changes in the intervention outcomes may have been influenced by contextual factors specific to the COVID-19 pandemic period. Plausible explanations lie in the changes brought about by the COVID-19 pandemic, such as heightened baseline stress for parents and increased behavioral challenges among children [34, 35]. Parents participating during the pandemic likely experienced elevated stress due to lockdowns, school closures, financial instability, and social isolation, which may have reduced their engagement with the intervention, thereby limiting its impact on improving child behavior [36]. This is evidenced by our data, which show an increasing trend in baseline parental DASS total scores over time, peaking during the COVID-19 period (Fig. S2). Furthermore, children enrolled during the pandemic may have exhibited higher baseline levels of externalizing problems due to disruptions in daily routines, limited social interactions, and increased family stress. Our findings indicate that baseline CBCL externalizing scores were highest for children enrolled in 2020–2021 compared to earlier enrollment periods, suggesting that preexisting behavioral difficulties may have contributed to the observed intervention effects (Fig.S3).

### Limitations

While the study has several strengths, some limitations should be acknowledged. A key limitation is the absence of a control group, which makes it difficult to attribute changes solely to the intervention. The observed changes in outcomes may reflect the natural course of children’s development, regression to the mean, or other time-related factors rather than the intervention itself. Nevertheless, the prior RCT of the same intervention reported sustained improvements over a two-year follow-up, supporting its efficacy. Second, the generalizability of our findings may be limited by the demographic characteristics of the sample, as the implementation was conducted in Finland, where the population is predominantly White and well-educated. Third, the outcome was assessed by parent reports, which may introduce reporting bias due to potential variations in parental interpretations of behavioral improvements. Additionally, families lost to follow-up were more often characterized by younger parental age, lower parental education, non–two-biological-parent family structure, greater initial child externalizing problems, harsher parenting practices, and poorer parental mental health. This attrition may underrepresent higher-risk families, potentially biasing the observed changes in outcomes and underestimating associations with parental characteristics. These findings should therefore be interpreted with caution, whilst future studies should aim to reduce dropout among higher-risk families to ensure more representative and robust results.

## Conclusion

This study explored various parental factors as predictors of changes in the outcome of the FSFP intervention, an internet-based and telephone-assisted parent training intervention. Effects of the intervention were predicted by maternal education, harsh or over-reactive parenting, and parental mental health over time. Overall, this intervention appears to be more beneficial for less-advantaged groups, including mothers with lower education, parents exhibiting harsher parenting styles, and parents with more severe symptoms of depression, anxiety, and stress. These findings may inform clinical practices for child disruptive behavior and guide the development of personalized digital interventions. Future research should also explore whether factors related to the child (e.g., duration or severity of disruptive behavior) or the intervention (e.g., completion rates, engagement, and time spent) serve as predictors or mediators of the intervention outcomes, providing valuable perspectives for enhancing the effect of such interventions.

## Supplementary Information

Below is the link to the electronic supplementary material.

Supplementary Material 1

## Data Availability

The data that support the findings of this study are not publicly available due to ethical and legal restrictions related to participant confidentiality. Access to the data is restricted by Research Centre for Child Psychiatry, University of Turku and cannot be shared.
