# Correction: Parental predictors of an Internet-based parenting intervention for child disruptive behavior: an implementation study

**DOI:** 10.1007/s00787-026-03020-8

**Published:** 2026-04-08

**Authors:** Yujing Li, Amit Baumel, Susanna Hinkka-Yli-Salomäki, Malin Kinnunen, Terja Ristkari, Minja Westerlund, Andre Sourander

**Affiliations:** 1 Department of Child Psychiatry, Research Centre for Child Psychiatry, University of Turku, Lemminkäisenkatu 3a, Turku, 20014 Finland; 2 Finland INVEST Research Flagship, University of Turku, Turku, Finland; 3 Department of Community Mental Health, University of Haifa, Haifa, Israel; 4 Department of Child Psychiatry, Turku University Hospital, Turku, Finland

**Correction: European Child & Adolescent Psychiatry**

10.1007/s00787-025-02928-x

In the original version of this article, the fig 1 appeared incorrectly in the online version.

The original PDF was correct.

