# Supplementary material for: Parental predictors of an Internet-based parenting intervention for child disruptive behavior: an implementation study
Source: Eur Child Adolesc Psychiatry. 2025 Nov 28;35(4):1257–66. doi: 10.1007/s00787-025-02928-x (PMC13219190; doi:10.1007/s00787-025-02928-x)
Supplement: Supplementary file 1 — Supplementary Material 1 [file 787_2025_2928_MOESM1_ESM.pdf]

Appendices

European Child & Adolescent Psychiatry

Parental Predictors of an Internet-based Parenting Intervention for Child Disruptive Behavior: An Implementation Study

Yujing Li[a,b*], Amit Baumel[c], Susanna Hinkka-Yli-Salomäki[a,b], Malin Kinnunen[a,b], Terja Ristkari[a,b], Minja Westerlund[a,b], Andre Sourander[a,b,d]

[a]Department of Child Psychiatry, University of Turku, Turku, Finland; [b]Finland INVEST Research Flagship, University of Turku, Turku, Finland; [c]Department of Community Mental Health, University of Haifa, Haifa, Israel; [d]Department of Child Psychiatry, Turku University Hospital, Turku, Finland

* Phone: +358 417 046 734 | Email: yujili@utu.fi | Address: Research Centre for Child Psychiatry, University of Turku, Lemminkäisenkatu 3a, 20014 Turku, Finland

Acknowledgments: This work was supported by the European Research Council under Grant 101020767 and the Research Council of Finland under Grant 320162.

The Finnish Strongest Families Parenting (FSFP) intervention is an internet-based, telephone-assisted parent training program targeting disruptive behavior in preschool children. The program is based on social learning theory, consisting of 11 sessions designed to enhance parenting skills to strengthen parent–child relationships, encourage positive behavior, manage daily routines, prepare for challenging situations, reduce conflict, and promote prosocial behavior.

A randomized clinical trial was conducted between October 2011 and November 2013. 464 families with 4-year-old children who screened positive for high levels of disruptive behavior at 42 child health clinics across 7 administrative regions in Southwest Finland and Satakunta were randomized to either the intervention (n = 232) or an educational control group (n = 232). The intervention group consistently showed greater improvements in child externalizing problems over the 24-month follow-up compared to the educational control group.

Since 2015, the FSFP intervention has been continuously implemented in Finland and has demonstrated substantial effectiveness. Information on the inclusion and exclusion criteria, intervention procedures and components, as well as quality measures and implementation plan, is described below.

Inclusion and exclusion criteria

This inclusion criteria for the implementation study were: (1) children had a score of ≥5 on the conduct problems subscale of the Strengths and Difficulties Questionnaire; (2) Parents reported at least minor difficulties in their child's emotions, behavior, or social skills; (3) Families had to reside in a participating administrative region; (4) At least one parent spoke Finnish or Swedish as their first language; and (5) Families possessed access to a phone, computer, and had Internet connection.

Children were excluded from the study if they: (1) were unable to speak in full sentences; (2) had hearing or vision impairments; (3) were receiving or had previously received behavioral interventions; (4) had a diagnosis of autism, Down syndrome, fetal alcohol syndrome, intellectual disability, severe mental disorders (e.g., psychosis or depression), or a genetic condition associated with mental retardation; (5) were living apart from their parents due to child protection services related to custody, abuse, or neglect; (6) fell outside the age range (i.e., younger than 3 years 6 months or older than 4 years 5 months). For families with

eligible twins, only the child exhibiting more severe problems was included, while the other twin was excluded.

Intervention Procedures and Components

The FSFP program integrates an interactive website with weekly telephone coaching. Coaches, primary healthcare or social service professionals such as public health nurses and social workers, guided the parents throughout the program. They conducted weekly telephone consultations with parents and continuously monitored their progress via the program website. The program consisted of 11 weekly themes, delivered through both the interactive website and assisted telephone coaching sessions. After the baseline questionnaire, coaches conducted an introductory call to present the program and the first theme, as well as to collaboratively set personalized goals with the parents for addressing specific child behavior issues. The first 7 sessions focused on teaching positive and practical parenting strategies, including positive problem-solving skills and understanding the child's emotional development. Later sessions focused on applying these skills in daily life and maintaining them after the program. The content and the framework of the weekly themes are depicted in Table S1. Each session included an introduction to the weekly theme, instructional content, video exercises, troubleshooting tips, and a summary of key takeaways. Videos and audio clips demonstrated how to apply the new skills, and coaches provided feedback and encouragement. Parents were only introduced to the next theme once they had completed the current one. Children were not involved in the web interactions or coaching calls.

Table S1. The structure of the Finnish Strongest Families Parenting program—an internet-based and telephone-supported parent training intervention

Weekly theme	Goals	Parental skills
Introduction to the program	Set up parents for success to actively start noticing the good in their child	
Notice the good	Guide parents on how to break the negative cycle and shift their perspective of the child	Notice the good in the child<br>Active parenting
Spread attention around	Teach parents to notice the good by spreading attention around<br>Strengthen child's friendship- and empathy skills	Impartial parenting<br>Learn to spread attention around actively<br>Praise the child for interacting positively with others
Ignore whining and complaining	Teach parents self-regulation and consistency	Use positive thinking to stay calm and in control of the situations
Prepare for changes	Reinforce good daily routines by preparing the child for upcoming changes and increase their listening	Use change warnings to prepare the child for changes<br>Use positive "when-then" statements

Weekly theme	Goals	Parental skills
Plan ahead at home	Emphasize the child's active participation and include them in the planning process for daily situations	Listen to the child’s ideas and plan daily activities at home
Reinforce by rewarding	Engage the child in planning and strengthen positive daily routines	Learn to set realistic goals and effectively use praises and rewards
Plan ahead outside the home	Emphasize the child’s active participation and include them in the planning process for situations outside the home	Listen to the child’s ideas<br>Plan situations outside the home
Cooperate with day care	Help child to manage and succeed in day care	Set realistic goals and rewards<br>Cooperate with day care
Plan how to use time-out	Teach parents self-regulation and consistency	Develop consistency and self-regulation in parental actions<br>Create strategies for managing challenging situations
Revise: Problem-solving and future application of skills	Encourage parents to use positive proactive parenting techniques	Recognize how applying skills can help avoid setbacks

Quality Assurance and Implementation Plan

To ensure data accuracy and intervention integrity, three core quality assurance components were implemented. First, coaches were carefully selected, extensively trained, and regularly supervised to maintain protocol adherence. Second, ongoing supervision, self-performance evaluations, and recorded phone calls (10% evaluated) were conducted to assess competency and ensure fidelity. Additionally, the FSFP intervention was centralized at the Research Center for Child Psychiatry at University of Turku, with regular meetings and progress reports to integrate the program into primary health care, supported by local and national media campaigns to increase public awareness of the program.

The dissemination of the program has been supported by both internal and external implementation strategies. Internal strategies focused on maintaining fidelity and effectiveness, including the use of a structured web-based platform for parents and coaches, assessments at baseline and at 6, 12, and 24 months, standardized training for all coaches, ongoing support for coaching via a coaches’ corner, quality assurance through recorded phone calls, and supervision with feedback. External strategies aimed at wider dissemination, involving integration into primary care within wellbeing services counties, close collaboration with decision-makers, regular training of healthcare nurses to strengthen early identification of families in need, provision of feedback on intervention use and outcomes, national seminars on child mental health, and use of public communication channels (e.g., newspapers, posters) for outreach.

Table S2. Baseline characteristics of the total sample and comparisons between completers and drop-outs.

	FSFP implementation (n = 2900)	Completed 24-month follow-up (n = 2107)	Did not complete 24-month follow-up (n = 793)	P value
**Child characteristics**				
**Sex[a], n (%)**				
Male	1828 (63.3)	1323 (63.0)	505 (36.0)	.638
Female	1062 (36.7)	778 (37.0)	284 (64.0)	
**Baseline CBCL externalizing score, mean (SD)**	21.7 (0.1)	21.4 (7.2)	22.5 (7.6)	<.001
**Parent and family characteristics**				
**Maternal age at birth[b], mean (SD)**	30.4 (5.0)	30.7 (4.9)	29.8 (5.3)	<.001
< 25 years, n (%)	345 (12.0)	202 (7.0)	143 (18.3)	<.001
25 – 35 years, n (%)	2090 (72.6)	1562 (54.3)	528 (67.5)	
> 35 years, n (%)	442 (15.4)	331 (11.5)	111 (14.2)	
**Paternal age at birth[c], mean (SD)**	32.6 (5.7)	32.8 (5.5)	32.1 (6.1)	.013
< 25 years, n (%)	189 (6.8)	111 (5.5)	78 (10.5)	<.001
25 – 35 years, n (%)	1805 (65.1)	1350 (66.5)	455 (28.2)	
> 35 years, n (%)	777 (28.1)	568 (28.0)	209 (61.3)	
**Maternal education[d], n (%)**				
Secondary education or less	1036 (35.9)	655 (31.2)	381 (48.5)	<.001
College or university degree	1851 (64.1)	1446 (68.8)	405 (51.5)	
**Paternal education[e], n (%)**				
Secondary education or less	1427 (52.3)	980 (49.0)	447 (61.4)	<.001
College or university degree	1302 (47.7)	1021 (51.0)	281 (38.6)	
**Family structure[f], n (%)**				
Two biological parents	2389 (82.7)	1783 (84.9)	606 (76.8)	<.001
One biological parent and other structures	500 (17.3)	317 (15.1)	183 (23.2)	
**Enrollment year, n (%)**				
2015-2017	707 (24.4)	510 (24.2)	197 (24.8)	<.001
2018-2019	1081 (37.3)	833 (39.5)	248 (31.3)	
2020-2021	1112 (38.3)	764 (36.3)	348 (43.9)	
**Parenting Scale Laxness score[g], n (%)**				
Low	1020 (35.2)	749 (35.6)	271 (34.2)	.346
Medium	1094 (37.7)	803 (38.1)	291 (36.8)	
High	785 (27.1)	555 (26.3)	230 (29.0)	
**Parenting Scale Over-reactivity score[h], n (%)**				
Low	856 (29.5)	594 (28.2)	262 (33.1)	.035
Medium	1147 (39.6)	846 (40.1)	301 (38.0)	
High	896 (30.9)	667 (31.7)	229 (28.9)	
**Parenting Scale Hostility score[i], n (%)**				
Low	1377 (47.5)	1036 (49.2)	341 (43.1)	.004
Medium	676 (23.3)	489 (23.2)	187 (23.6)	
High	846 (29.2)	582 (27.6)	264 (33.3)	
**DASS Total score[j], n (%)**				
Low	1096 (37.8)	836 (39.7)	260 (32.8)	.003
Medium	1153 (39.8)	814 (38.6)	339 (42.8)	
High	650 (22.4)	457 (21.7)	193 (24.4)	

Note. FSFP = Finnish Strongest Families Parenting; SD = standard deviation; DASS = the 21-item short form of the Depression, Anxiety and Stress Scale.
[a]Missing observations: for total sample n = 10, for completers n = 6, for drop-outs n = 4;
[b]Missing observations: for total sample n = 23, for completers n = 12, for drop-outs n = 11;
[c]Missing observations: for total sample n = 129, for completers n = 78, for drop-outs n = 51;
[d]Missing observations: for total sample n = 13, for completers n = 6, for drop-outs n = 7;
[e]Missing observations: for total sample n = 171, for completers n = 106, for drop-outs n = 65;
[f]Missing observations: for total sample n = 11, for completers n = 7, for drop-outs n = 4;
[g]Missing observations: for total sample n = 1, for drop-outs n = 1;
[h]Missing observations: for total sample n = 1, for drop-outs n = 1;
[i]Missing observations: for total sample n = 1, for drop-outs n = 1;
[j]Missing observations: for total sample n = 1, for drop-outs n = 1.

Fig. S1 Correlation of the potential predictors

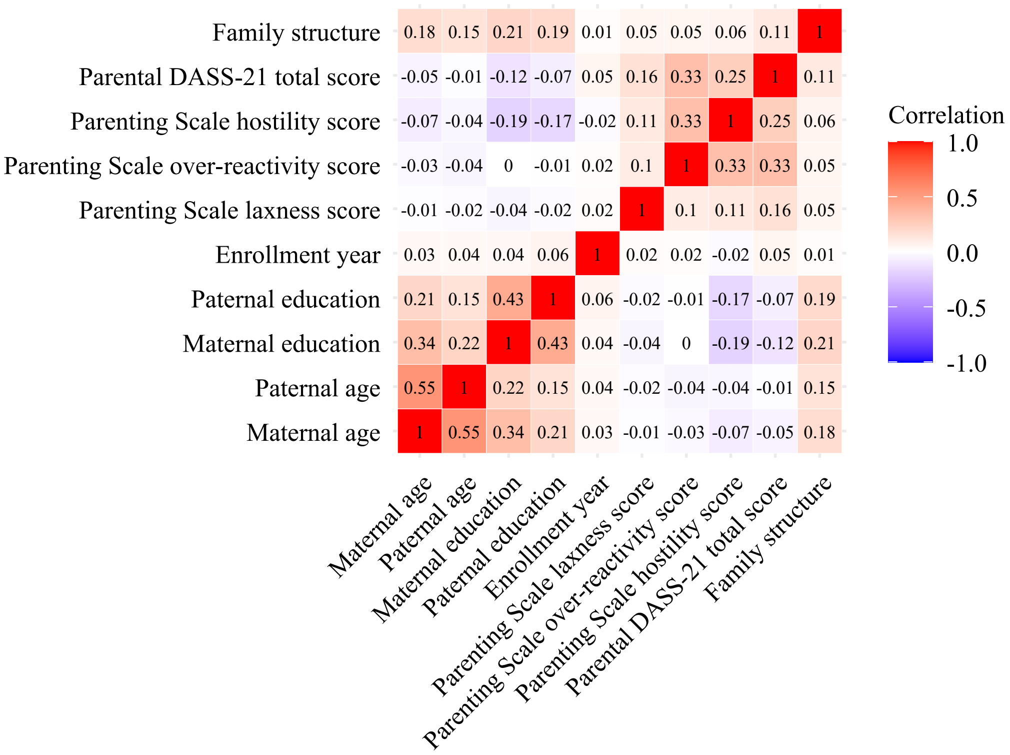

Note. Spearman's rank correlation and Cramér’s V were used for calculating the correlations between ordinal-ordinal and nominal-ordinal variables, respectively. Ordinal variables include maternal age, paternal age, maternal education, paternal education, enrollment year, Parenting Scale laxness, over-reactivity, and hostility scores, and Parental DASS-21 total score, while family structure is a nominal variable. DASS-21 = the 21-item short form of the Depression, Anxiety and Stress Scale.

Fig. S2 Average baseline parental DASS total score across the enrollment period

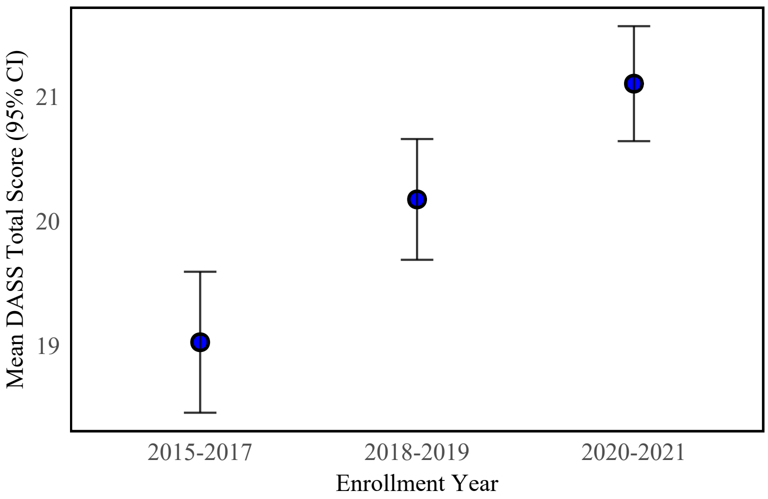

Note. DASS = the 21-item short form of the Depression, Anxiety and Stress Scale. 95% CI = 95% confidence interval.

Fig. S3 Average baseline CBCL externalizing score across the enrollment period

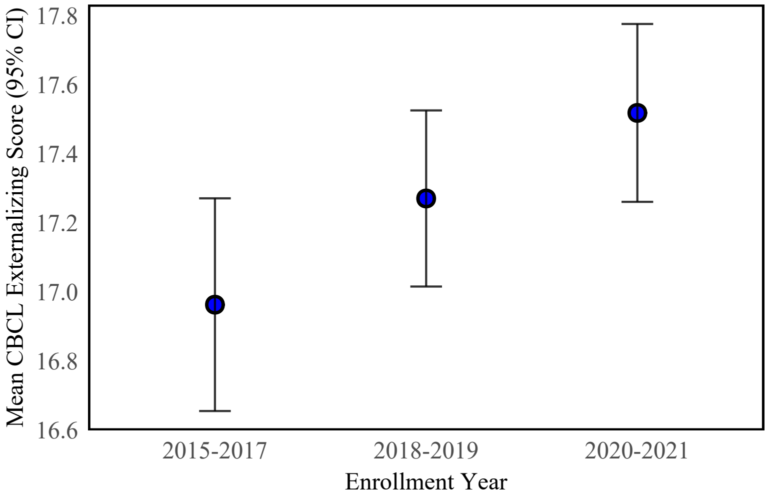

Note. CBCL = Child Behavior Checklist 1.5-5. 95% CI = 95% confidence interval.
